# The Possible Role of Cancer Stem Cells in the Resistance to Kinase Inhibitors of Advanced Thyroid Cancer

**DOI:** 10.3390/cancers12082249

**Published:** 2020-08-11

**Authors:** Fiorenza Gianì, Veronica Vella, Dario Tumino, Pasqualino Malandrino, Francesco Frasca

**Affiliations:** Endocrinology, Department of Clinical and Experimental Medicine, University of Catania, Garibaldi-Nesima Medical Center, 95122 Catania, Italy; fiorenza.giani@gmail.com (F.G.); veronica.vella@unict.it (V.V.); dario.tumino@phd.unict.it (D.T.); p.malandrino@unict.it (P.M.)

**Keywords:** thyroid cancer, stem cells, kinase inhibitors, ERK pathway, tumor relapse

## Abstract

Target therapy with various kinase inhibitors (KIs) has been extended to patients with advanced thyroid cancer, but only a subset of these compounds has displayed efficacy in clinical use. However, after an initial response to KIs, dramatic disease progression occurs in most cases. With the discovery of cancer stem cells (CSCs), it is possible to postulate that thyroid cancer resistance to KI therapies, both intrinsic and acquired, may be sustained by this cell subtype. Indeed, CSCs have been considered as the main drivers of metastatic activity and therapeutic resistance, because of their ability to generate heterogeneous secondary cell populations and survive treatment by remaining in a quiescent state. Hence, despite the impressive progress in understanding of the molecular basis of thyroid tumorigenesis, drug resistance is still the major challenge in advanced thyroid cancer management. In this view, definition of the role of CSCs in thyroid cancer resistance may be crucial to identifying new therapeutic targets and preventing resistance to anti-cancer treatments and tumor relapse. The aim of this review is to elucidate the possible role of CSCs in the development of resistance of advanced thyroid cancer to current anti-cancer therapies and their potential implications in the management of these patients.

## 1. Introduction

The incidence of thyroid cancer has steadily increased over the past decades, with an annual percent change of +4.7 for both genders in the period between 2004 and 2013 [1]. Although this increased incidence may be due to improved diagnostic scrutiny [2], some evidence supports a real increase. Indeed, along with small tumors, both larger (i.e., >4 cm) and metastatic tumors have also increased. As a consequence, thyroid cancer-related mortality has also increased in the United States [3]. Hence, the burden of possible carcinogenic factors has been hypothesized, though, to date, the only well-recognized factors are radiation exposure, low-iodine diet and family history of thyroid cancer [4,5]. Approximately 95% of thyroid cancers, classified as differentiated thyroid cancers (DTCs), have an excellent prognosis with a 10-year survival rate of 85% [6]. Indeed, surgery and radioiodine are effective for the large majority of patients with thyroid cancer, even for those with a tumor relapse (at local or distant sites). However, some patients with distant metastases are not responsive to radioiodine treatment and their 10-year survival rate falls to about 10% [7]. Moreover, poorly differentiated thyroid cancer (PDTC) and anaplastic thyroid cancer (ATC) that are usually diagnosed at an advanced stage, with local invasion of the soft tissue of the neck and distant metastasis, also fail to uptake radioactive iodine (RAI). About 5% and 2% of all thyroid cancers (PDTC and ATC, respectively), because of their aggressive clinical behavior and intrinsic resistance to RAI and other therapeutic approaches, are responsible for 40–50% of thyroid cancer-related death [8]. For several decades, therapeutic options for patients with metastatic thyroid cancer have been limited to systemic chemotherapy, such as doxorubicin alone or in combination. The response rate (i.e., including patients with partial and complete responses) to these regimens is about 20–25%, with median survival of approximately 18 months in advanced DTC [9]. The use of new agents targeting specific molecular pathways has only partially met the medical needs of patients with advanced progressive thyroid cancer in recent years [10,11,12]. Sorafenib and lenvatinib are the most used multi-tyrosine kinase inhibitors (MKIs) approved by the Food and Drug Administration (FDA) and the European Medicines Agency (EMA) for the treatment of locally recurrent or metastatic, progressive DTC refractory to RAI treatment. Sorafenib was approved based on the results of a phase 3 multicenter study that documented an improvement in the progression-free survival (PFS) of five months compared to placebo (10.8 vs. 5.8 months) [13]. The efficacy of lenvatinib was documented by a multicenter study showing an improvement in the PFS of 15 months compared to placebo (18.3 vs. 3.6 months) [14]. Despite these promising results, both drugs are associated with severe grade 3–4 adverse events, including hand-foot skin reaction, hypertension, diarrhea, fatigue and weight loss. Moreover, within 18 months from the initiation of sorafenib and lenvatinib, half of the patients displayed a progressive disease. While in patients with PDTC and ATC, unfortunately, most MKIs have shown limited or negligible activity, and the combination of two targeted drugs, dabrafenib and trametinib, is the one FDA-approved treatment for the patients with BRAF V600E mutation-positive ATC.

The mechanisms of this resistance have been partially elucidated and include the up-regulation of multidrug resistance and antiapoptotic proteins [15,16,17] and the activation of pro-angiogenic signaling pathways [18]. However, the majority of biological mechanisms leading to impressive tumor dedifferentiation, aggressiveness and relapse remain to be unraveled to date.

Recent experimental evidence has demonstrated the presence of a small subpopulation of cells within the tumor mass, namely cancer stem cells (CSCs), which may play a role in tumor initiation, growth and metastatic spread [19]. These CSCs, which may be functionally considered DNA packaging cells due their ability to arrest in G0, are very resistant to the genotoxic effect of chemotherapy [20].

In this regard, we reviewed the possible role of CSCs in promoting progression and resistance to current anti-cancer treatment in advanced and PDTCs.

## 2. Thyroid Tumor Features

### 2.1. Thyroid Cancer Histotypes

The thyroid gland may give rise to several histological tumor types and subtypes that differ in cellular shape, molecular profile, biological behavior and prognosis. Based upon histopathological features, thyroid cancer is divided into four main subtypes: papillary; follicular; anaplastic, which is follicular-derived thyroid cancer; and medullary thyroid cancer (MTC), which originates from the neuroendocrine C-cells. Papillary (PTC) and follicular (FTC) thyroid cancers are usually classified as differentiated thyroid cancers (DTCs), as they maintain several histological features of follicular cells. DTC is three times more frequent in women than in men and has a peak incidence in the third and fourth decades [1]. Approximately 90% of all thyroid cancers are PTCs, with a good overall prognosis and spread to loco-regional lymph nodes (and less frequently to the lungs). The second most common subtype is FTC, which represents 5% of all thyroid cancers and metastasizes by bloodstream to distant sites, especially to lung and bone. DTCs often retain the ability to entrap iodine and are therefore usually sensitive to RAI treatment. PDTC is histologically distinct from well-differentiated thyroid carcinoma and displays aggressive behavior, which is intermediate between differentiated and undifferentiated thyroid cancers. Anaplastic thyroid cancer (ATC) is one of the most aggressive and deadly cancers in humans. This rare cancer type (<1% of thyroid cancer) displays a peak onset at around 65 years or older [1]. At the time of diagnosis, many patients already have advanced disease, including a large cervical expanding mass that requires tracheostomy to maintain airway patency, and distant metastases (lungs, bones and brain), which determine a short survival (approximately 5–6 months) [21]. Furthermore, MTC is a rare thyroid tumor, accounting for 1–2% of all thyroid cancers [1]. It is derived from para-follicular C-cells, capable of secreting calcitonin and carcinoembryonic agent (CEA), which represent clinically useful markers for diagnosis and follow-up. This tumor is typically more aggressive than DTC, spreads to lymphatic and blood vessels and metastasizes to cervical lymph nodes, lungs and liver.

### 2.2. Genetics of Thyroid Cancer

DNA sequencing studies have identified the most relevant genetic alterations in thyroid carcinomas. Several of these mutations occur in genes with kinase activity, which are involved in the mitogen-activated protein kinase (MAPK), the phosphatidylinositol 3-kinase (PI3K) pathways or both. These signaling cascades transmit growth signals to the nucleus and their aberrant activation has been implicated in the onset, progression and aggressiveness of thyroid cancer. The increasing knowledge about these molecular alterations has allowed the development of new drugs targeting these pathways, such as multi-tyrosine kinase inhibitors (MKIs) [22].

#### 2.2.1. Differentiated Thyroid Cancer (DTC)

The Cancer Genome Atlas (TCGA) program [23] has recently completed the whole genomic analysis in about 500 PTCs. These studies largely confirmed previous data about the frequency of key mutations in this disease, mostly belonging to the MAPK signaling pathway. In addition, many novel mutations were discovered, for example, BRAF mutations other than BRAFV600E, novel RET partners, many forms of BRAF fusions and BRAF indels [24]. In PTCs, TGCA identified BRAF mutations in 61.7% of cases, RAS mutations in 12.9%, RET fusions in 6.8% and BRAF fusions in 2.7% [24]. These alterations are nearly mutually exclusive, supporting the concept of the absence of a positive selective pressure toward progressive DNA damage accumulation in a single cell. These data support the idea that these aberrant genes encode for proteins operating in convergent pathways [25]. The most frequent mutation in DTC is the BRAF T1799A mutation, resulting in a mutant BRAF V600E kinase, present in PTC and papillary-derived ATC. Mutations in the RAS family of oncogenes are frequent in FTC and the follicular variant of PTC.

As BRAF and RAS mutants are the predominant driver of oncogenes, they determine two different signaling patterns and tumor phenotypes. Tumors with true papillary architecture are characterized by BRAFV600E mutations and RET fusions and are classified as “BRAFV600E-like”, which display higher MAPK transcriptional output and lower expression of genes involved in iodine metabolism. On the other hand, tumors with follicular architecture are characterized by RAS mutations and classified as “RAS-like”, with a lower MAPK signaling output and relatively preserved expression of iodine-related genes [24]. Thirty percent of FTCs harbor chromosomal translocations resulting in a fusion gene between the thyroid transcription factor PAX8 gene and the PPARγ gene (PAX8-PPARγ) [26], while RET oncogene rearrangements are present in about 7% of PTCs [23]. BRAF mutations have been involved in a greater clinical aggressiveness, as well as TERT mutations, that are found in 7.5% of PTCs, 17.1% of FTCs, 29.0% of PDTCs and 33.3% of ATCs [27].

#### 2.2.2. Poorly Differentiated Thyroid Cancer (PDTC) and Anaplastic Thyroid Cancer (ATC)

TCGA genomic study in PTCs, along with some recent next-generation sequencing studies performed in PDTCs and ATCs, support a gradual tumor progression from well-differentiated to poorly differentiated and ultimately to ATCs through the accumulation of key additional genetic alterations. While RAS and BRAF V600E mutations remain the main drivers in PTCs and PDTCs, other mutations such as TERT and TP53, as well as alterations of the PI3K/AKT pathway are more frequent in ATC [28].

#### 2.2.3. Medullary Thyroid cancer (MTC)

Most MTCs display a mutation in the RET proto-oncogene, while rarely H-RAS, K-RAS or N-RAS mutation [29]. RET mutation may be either sporadic or germline, with autosomal dominant inheritance. There are three familial patterns: multiple endocrine neoplasia 2 A (MEN 2A), MEN 2B and familial medullary cancer, without any association with other endocrine tumors (FMTC) [30].

## 3. The Multi-Step Carcinogenesis Model

**Figure 1:**
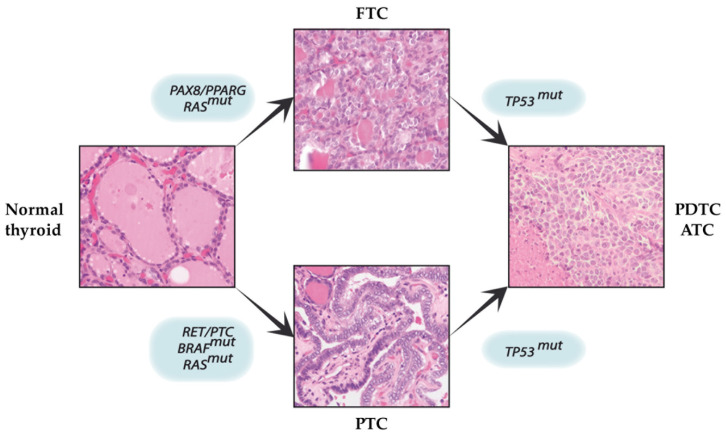
Schematic representation of multistep carcinogenesis in thyroid cancer. This model suggests a stepwise dedifferentiation process from mature thyroid cells, to well-differentiated cancer, up to poorly differentiated and anaplastic thyroid cancer, as the result of progressive accumulation of somatic genetic alterations (H&E stain, original magnification: 200×). Abbreviations: FTC = follicular thyroid cancer, TP53 = tumor protein p53, PAX8/PPARG = paired box 8/peroxisome proliferator-activated receptor gamma fusion protein, RAS^mut^ = rat sarcoma viral oncogene homolog mutations, RET/PTC = rearranged during transfection/papillary thyroid carcinoma fusion protein, BRAF^mut^ = v-raf murine sarcoma viral oncogene homolog B1 mutations, PTC = papillary thyroid cancer, TP53^mut^ = tumor protein p53 mutations, PDTC = poorly differentiated thyroid cancer, ATC = anaplastic thyroid cancer, H&E stain = hematoxylin and eosin stain.

Along with other malignances, experiments based upon the exposure to chemicals led to hypothesizing the multi-step carcinogenesis model in thyroid cancer. According to this model, thyroid cancer cells arise from the gradual accumulation of genetic alterations within normal thyroid epithelial cells, leading to progressive acquisition of increased proliferation and an invasive phenotype [31] (Figure 1).

In this respect, mutations in both tumor suppressor genes and proto-oncogenes are crucial in the carcinogenesis model (driving mutations). As discussed above, a key mutation in PTC is V600EBRAF [32], and RAS point mutations are present in adenomas, follicular carcinomas and follicular variant of PTCs. Chromosomal rearrangements are also frequently found in DTCs [33] and RET proto-oncogene rearrangements and activation are present in early stages of PTCs, particularly common in patients exposed to ionizing radiation and in pediatric cancer [34]. On the other hand, PAX8-PPARγ rearrangement is important for the progression of follicular adenoma to follicular carcinoma [35]. According to this multi-step model, undifferentiated and poorly differentiated thyroid carcinomas originate from either dedifferentiated follicular or papillary cancers harboring inactivated tumor suppressors, such as p53 [36]. Nevertheless, this multi-step carcinogenesis model does not fully explain some clinical and molecular features of thyroid cancer (i.e., how the low proliferation rate of normal thyrocytes may support the increased thyroid cancer risk in adults?). Moreover, if ATCs derive from DTCs by accumulation of further genetic abnormalities, why are BRAF mutations and RET/PTC and PAX8/PPARγ rearrangements typical in DTCs seldom present in ATCs? Hence, the multistep carcinogenesis model does not fully fit into the development of a consistent number of PDTCs.

## 4. A New Paradigm in TC Origin and Progression: From the Multi-Step Carcinogenesis Model to the Cancer Stem Cell (CSC) Hypothesis

Development of either pre-existing or acquired resistance is based upon either the presence or progressive accumulation of specific molecular alterations in thyroid cancer (TC) cells. Identifying molecular abnormalities that underlie tumor onset and progression may be helpful to better depict the multi-step carcinogenesis model and also analyze those mechanisms responsible for drug resistance.

On the other hand, several studies, indicating that the cancer cell population is heterogeneous and molecular alterations are not present in the whole tumor bulk, were allowed to depict the “cancer stem cell hypothesis”. This hypothesis was firstly established by the previous observation that leukemia may contain hierarchical multi-lineage cells [37]. In this view, it is reasonable to suppose that a subpopulation of tumor cells may be involved in the pathogenesis, progression and evolution of human solid tumors. Several observations also indicate that these cancer cell subtypes share many properties with stem cells, including self-renewal and indefinite growth [38,39].

**Figure 2:**
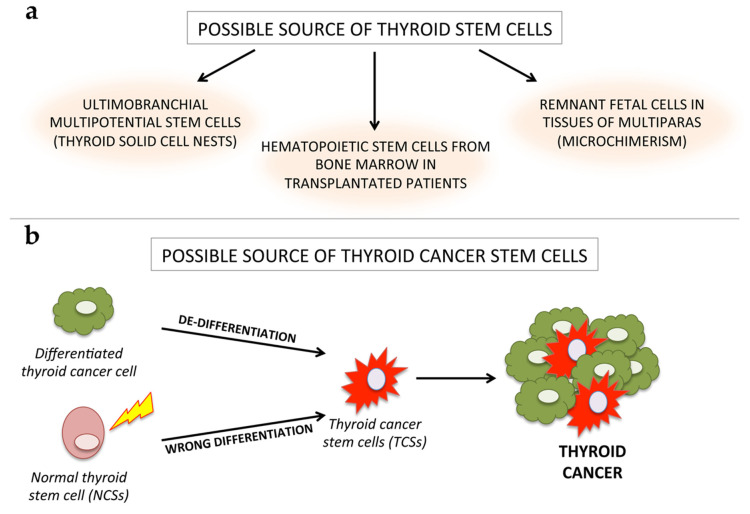
The possible origin of thyroid stem cells (SCs). (**a**) Different hypotheses about the origin of thyroid SCs: (1) from ultimobranchial body cell nests; (2) from stem cells migrating from different tissues (e.g., bone marrow) and (3) from remnant fetal cells present in tissue of multiparas. (**b**) TCSs may originate either from transformation of normal thyroid stem cells or through de-differentiation of thyroid cancer cells which assume CSC characteristics. According to this model, CSCs are a key component of tumor heterogeneity.

In this perspective, some authors hypothesized that thyroid cancer may be a cancer stem cell driven disease [40,41,42]. A debate about the origin of thyroid stem cells in adult human thyroid is still open and several possible sources have been hypothesized [43,44] (Figure 2a).

The presence of adult stem/progenitors within the thyroid gland was first postulated by Dumont who estimated the theoretical number of 1 stem cell every 1000 mature thyroid follicular cells [45]. Later, immunohistochemical studies indicated the presence of a cell population within thyroid tissue positive for stem cell markers, such as octamer-binding transcription factor 4 (Oct-4), GATA Binding protein 4 (GATA-4) and hepatocyte nuclear factor 4 alpha (HNF4α) [46]. A possible source of thyroid stem cells was postulated by Harach, who described the presence of ultimobranchial body nests in fetal thyroid tissue displaying a similarity with adult thyroid solid cell nests (SCNs) [47]. A possible role of ultimobranchial multipotential stem cells in the histogenesis of thyroid carcinomas, such as mucoepidermoid thyroid carcinoma, was first evidenced in 1995 [48] and later on [49]. A further source of thyroid stem cells may be allogenic, from remnant fetal cells present in tissues of multiparas. This phenomenon is called microchimerism and is due to the bidirectional trafficking and persistence of a small number of allogenic cells in genetically different organisms. Hence, these fetal cells could be a possible alternative source of thyroid stem/progenitor cells. Although they have been detected in normal thyroid tissues and follicular adenomas in multiparas [50], their role in cancer stem cell origin has not yet been demonstrated.

Finally, the observation that patients who received hematopoietic stem cell transplantation have an increased risk of thyroid cancer led to the hypothesis that hematopoietic stem cells from bone marrow may migrate to thyroid tissue and reside there for a long time [51]. However, with respect to the thyroid gland, it was difficult to prove the existence of stem cells, with proliferation and differentiation abilities in adult [46,52]. Several approaches to isolate thyroid stem cells were attempted, but to date, no standardized method has been established. The first method to isolate TSCs was performed by generating thyrospheres from adult thyroid tissue. These thyrospheres were obtained by the exposure of single thyroid cell suspension to growth factor stimulation (bFGF and EGF) in serum-free medium and non-adherent culture conditions. Exposure of these thyrospheres to serum and TSH induced differentiation into thyrocytes [53], while the addition of insulin, insulin-like growth factor (IGF) or both, stimulated the growth of TSCs and thyrosphere volume [54]. Thyroid stem cells are supposed to be involved in tissue regeneration and repair after injury, such as thyroid follicle proliferation after partial thyroidectomy in mice [55]. Three types of thyroid stem cells have been described: the progenitor of follicular cells (of endodermal origin); the progenitor of C-cells, originating in the ultimo-branchial bodies (neural crest origin); and bipotential progenitors, giving rise to both follicular and C-cells, as indicated by immunofluorescence studies. The transition of stem cells into mature cells is stimulated by growth factors and cytokines present in the microenvironment of the stem niche.

According to this view, a given tumor may behave as an organoid containing both cancer stem cells and cancer differentiated cells. These CSCs may originate from either normal stem cells (NSCs) through a transformation process or from differentiated cancer cells as the result of a dedifferentiation process [40] (Figure 2b). The CSC model may have important implications for both the diagnosis and treatment of thyroid cancer, especially for the management of poorly differentiated, recurrent or rapidly growing diseases, refractory to RAI therapy.

**Figure 3:**
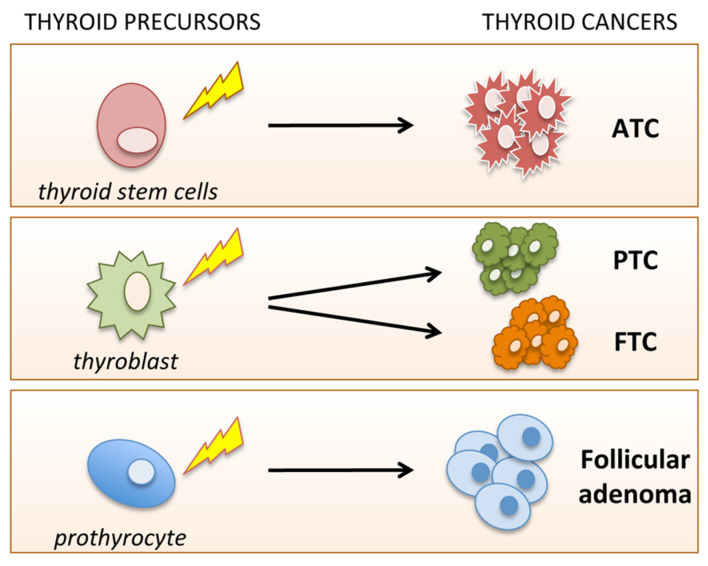
Schematic representation of fetal carcinogenesis in thyroid cancer. According to this model, thyroid cancer cells derive from the transformation of the three types of fetal thyroid cells, namely, thyroid stem cells, thyroblasts and prothyrocytes, which may give rise to ATC, PTC and FTC, and follicular adenoma, respectively.

The fetal cell carcinogenesis hypothesis in thyroid cancer previously proposed by Takano is in line with this notion. Indeed, he postulated an initial carcinogenetic event occurring in fetal thyroid cells, which are present during the fetal life until childhood [56]. This mutational event may occur in thyroid precursors at different stages of differentiation: thyroid stem cells (TSCs), thyroblasts and prothyrocytes, giving rise to the different TC histotypes. According to this view, ATC takes its origin from TSCs (expressing TTF-1 e NKX-1), PTC and FTC from thyroblasts (cells responsible for thyroid growth during the development expressing TG), and follicular adenomas from prothyrocytes (fetal thyroid cells that form follicles, but not still committed to produce thyroid hormone) [57] (Figure 3).

This hypothesis implies that the carcinogenetic event occurs in the early phases of thyroid development and the transforming potency of BRAF and RET oncogenes mostly depends on the inhibition of differentiation of thyroid precursors. On the other hand, this model is in accordance with the low proliferation rate of thyroid cells, the late appearance of the tumor in adult age and is partially supported by recent molecular studies [57,58,59,60,61]. The CSC model and multistep carcinogenesis model may be not mutually exclusive, but rather, they may coexist and explain the extreme variability in the behavior of TC. In this regard, it is reasonable to hypothesize that the “multistep carcinogenesis model” may be applied to adult stem cells [62] and to the “fetal cell carcinogenesis model” [57].

## 5. Normal and CSCs: Characteristics and Classification

To explain the possible involvement of thyroid cancer stem cells in chemoresistance, the peculiar features of both normal and cancer stem cells are described below. Normal stem cells display two hallmark properties: (a) self-renewal (i.e., the capacity to maintain the lineage by generating daughter cells that remain stem cells) and (b) potency (i.e., the ability to differentiate into specialized cells) [63]. As a consequence, the peculiar properties of stem cells are unlimited division in an un-differentiated state. In order to maintain a cell population lineage with the same characteristics, stem cells undergo two types of cell division: (a) symmetric division, generating two identical cells with stem cell features and (b) asymmetric replication, giving rise to one daughter cell identical to the mother and the other daughter cell undergoing a differentiation program. During both normal development and tumor progression, stem cell division and differentiation rates are influenced by the microenvironment, which plays a crucial role in stem cell biology and survival [64]. Stem cells may be classified as totipotent, when they differentiate into both embryonic and extraembryonic cell types; pluripotent, deriving from totipotent cells and differentiating into cell types belonging to the three germ cell layers (ectoderm, endoderm and mesoderm); multipotent may differentiate into a defined number of cell types, which are closely related to the original cell type; oligopotent can differentiate into only a few cell types, such as lymphoid or myeloid stem cells; and unipotent cells produce only a distinct cell type, maintaining the self-renewal capability. NSCs may be also classified as embryonic (ESCs), germinal and adult (somatic) stem cells (SSCs) with a different proliferative potential. SSCs or progenitor cells are limited in their proliferative and differentiation potentials lying in an undifferentiated state in the majority of adult tissues as quiescent cells that replicate themselves only for renewal or after injury [65]. Stem cells may be isolated from adult tissues by clonogenic methods in vitro that evaluate their ability of self-renewal, differentiation, and expression of molecular markers [46]. Different molecular pathways regulate stem cell self-renewal in different tissues, and the same pathway may be used by both NSCs and CSCs to regulate proliferation and differentiation. Studies of these common pathways may be crucial for the identification of molecular targets in anti-cancer therapies.

Similar to NSCs, CSCs are a subpopulation within a tumor mass with long-term self-renewal and the ability to generate a progeny differentiating into different tumor cells that variably contribute to tumor expansion [66]. After isolation, these CSCs are able to generate the same tumor phenotype when implanted into animal models [67]. Due to common features with NSCs, CSCs are resistant to the common anti-cancer treatments and are responsible for tumor relapse and metastasis. Indeed, traditional chemotherapy is usually able to kill cancer differentiated and proliferating cells, but not the slow dividing subpopulation of CSCs that is resistant and eventually sustains tumor relapse. Therefore, the emerging topic in anticancer research is the development of specific therapies targeted against CSCs, especially in those patients with advanced and relapsing disease. Tumor progression may be explained by these two main models: the multistep (or stochastic) and the stem cell (or hierarchical) model. According to the first model, tumor growth is sustained by each tumor cell while, according to the second, tumor cells are organized hierarchically along different subtypes, while a restricted subpopulation drives the overall tumor progression and growth. These two different theories are not mutually exclusive, as the plasticity of cancer may be based upon a transition from non-CSCs to CSCs and vice versa, in accordance with different conditions in the tumor microenvironment. Similar to NSCs, isolation of CSCs from tumor tissues, including thyroid cancers, have been performed by different approaches, including antigen-directed FACS sorting, side population and Aldefluor assay [52,68]. Although in vitro culture and maintenance of CSCs is difficult, the continuous development of new technologies has allowed the CSC culture as cell floating spheroids in non-adherent plates or 3D culture in reconstituted basal membranes (Matrigel) [69,70]. An important feature of CSCs, at various limiting dilution, is the ability to reproduce the original tumors in vivo. Compared to NSCs, CSCs express a specific set of cell markers and exhibit dysregulated signaling pathways and abnormal phenotypes. The dysregulated pathways in CSCs include Wnt, Hedgehog and Notch signaling, which are involved in self-renewal of NSCs. Recent evidence indicates that CSCs are involved in cancer cell epithelial to mesenchymal transition (EMT) and, as a consequence, in tumor invasiveness and distant metastasis. In this view, EMT arises from the generation of thyroid CSCs with mesenchymal cell properties [71]. With respect to thyroid cancer, Lan et al. found that the majority of ATC cells display an EMT phenotype and share biological and molecular features common to NSCs and CSCs. These authors also showed that the content of CSCs in the ATC cell population directly correlates with the presence of EMT. They concluded that the emergence of a CSC-like phenotype is a consequence of EMT onset in epithelial thyroid cancer cells. Experimental evidence indicates that hypoxia in the thyroid tumor microenvironment is able to activate the Wnt/β-catenin signals, thereby inducing the EMT program [72]. Further characterized CSC pathways include, Sonic hedgehog (Shh)/Patched (Ptch)/Smoothened (Smo) [73], Notch/Delta-like ligand (DLL) [74], CXC chemokine receptor 1–2/CXCL8/FAK [75] and Wnt [76]. These pathways may also modulate downstream effectors, including transcription factor activators and transcription factors, such as β-catenin, STAT3 [77] and Nanog [78,79]. Moreover, some reports indicate that undifferentiated phenotype and tumor aggressiveness in PTC is dependent on a selective upregulation of a set of genes in CSCs. This alteration is concomitant with the up-regulation of Wnt and Notch signaling and with increased EMT and chemoresistance [79]. Todaro et al. [61] demonstrated that activation of AKT, c-Met and β-catenin, along with downregulation of E-cadherin, confers motile and invasive behavior to undifferentiated thyroid CSCs. Similar to CSCs originating from other tumors, thyroid CSCs display high ALDH activity, along with increased clonogenic and self-renewal abilities [61]. Many other biomarkers have been proposed to distinguish thyroid CSCs from the bulk of tumor cells in order to facilitate their isolation and characterization in vitro. In PTCs, CD44- and ALDH-positive cells have been correlated to BRAF mutation and extrathyroidal extension in node-positive patients [80]. Further studies indicated that CXCR1 is expressed in PTC tissues, where it sustains an IL-8 autocrine loop that regulates thyroid CSC behavior. Moreover, there is evidence that CD34 is overexpressed in thyrospheres, which stimulates the sphere-forming ability and tumorigenicity of thyroid cancer cells [81]. Nevertheless, the urgent need to find functional molecules with a critical role in CSC survival and self-renewal, rather than simply markers, has led to the identification of specific signaling pathways increasing the possibility of developing clinical strategies. This is the case of the JAK/STAT3 and NF-kB pathways whose activation has been discovered to be able to confer CSC properties to ATC cells [82].

A growing body of evidence highlights the importance of the IGF system in the biological behavior of thyroid CSCs [54,83]. Several features of cancer cells, such as increased proliferation, stemness, metabolic reprogramming and resistance to therapies, are associated with IGF system activation [84,85,86,87]. Specifically, both IGF2 and the fetal A isoform of IR are frequently overexpressed in cancers, resulting in the activation of a loop that leads to a potentiation of cancer phenotype. In addition, the cross-talk of IR with its cognate receptor IGF-1R gives rise to the formation of hybrid receptors (HR-A or HR-B), further enhancing the IGF signaling in cancer cells. More recently, a functional cross-talk between IR-A and a non-integrin collagen receptor, discoidin domain receptor 1 (DDR1), has been described, adding more complexity to the system [88,89,90,91]. Nonetheless, the specific role of these pathways in PTC spheres and in thyroid tumor progression is still under investigation.

## 6. Role of CSCs in the Development of Resistance to Thyroid Cancer Treatments

**Figure 4:**
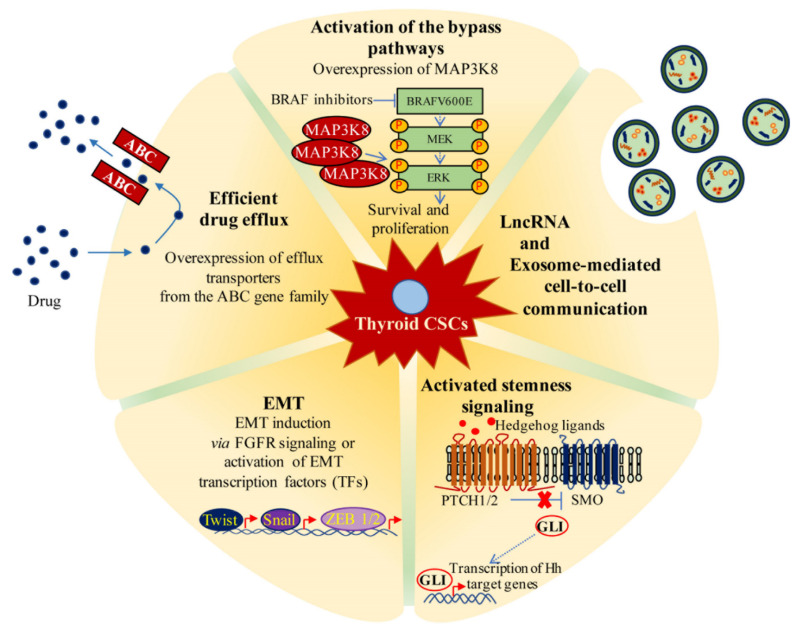
Molecular mechanisms underlying thyroid cancer stem cells (CSCs) resistance to therapy with kinase inhibitor. Resistance of CSCs to kinase inhibitor (KI) may be caused by different mechanisms, including epithelial-to-mesenchymal transition, increased drug efflux efficiency, activation of the bypass pathways, lncRNA and exosomes mediated cell-cell communication and deregulation of the stem cell signaling pathways. Abbreviations: MAP3K8 = Mitogen-Activated Protein Kinase Kinase 8, BRAF = v-raf murine sarcoma viral oncogene homolog B1, BRAFV600E = valine-to-glutamic acid substitution at amino acid residue 600 of BRAF protein, MEK = Mitogen-Activated Protein Kinase Kinase, ERK = Extracellular Signal-Regulated Kinase, LncRNA = Long non-coding RNA, PTCH1/2 = Patched1 and 2, SMO = Smoothened, GLI = glioma-associated oncogene family zinc finger, Twist = Twist-related protein 1, Snail = zinc finger protein SNAI1, ZEB *½* = Zinc finger E-box binding protein 1 and 2, TF = transcription factor, EMT = epithelial-mesenchymal transition, FGFR = fibroblast growth factor receptor.

Resistance to therapy continues to be the main challenge in achieving long-term remission or cure in patients with advanced thyroid cancer. Multiple underlying mechanisms have been identified over the past several years. For example, the mutations in downstream effector molecules of tyrosine kinase receptors [92,93], the activation of compensatory signaling pathways [94,95], the secondary acquired mutations [96,97,98] and the tumor microenvironmental features [99] are all potential causes of failures of molecular-targeted therapy. In the last two decades, attention has been focused on the role of CSCs. Current views favor the model that the differentiated cancer cells or non-CSCs undergo apoptosis after treatment with anti-cancer therapies, whereas the CSCs exhibit characteristics of therapy resistance, thereby escaping apoptotic cell death. Then, the remaining CSCs can re-establish tumor growth and cause relapse from therapies. Thyroid CSCs have been shown to be highly resistant to most of the available anti-cancer therapies, including conventional chemotherapy and radiotherapy. Currently, few findings have been reported implying that CSCs contribute to KI resistance in advanced thyroid cancer. The mechanisms by which the thyroid CSCs contribute to therapy resistance, with an emphasis on molecular-targeted inhibitors, are mentioned below in detail and summarized in Figure 4. Furthermore, we outlined some potential therapeutic strategies to overcome therapy resistance of thyroid CSCs.

### 6.1. CSCs: “The Survivors” to Conventional Anti-Cancer Therapy

Thyroid cancer is historically refractory to chemotherapy and radiotherapy. Since their identification and characterization in thyroid cancer, CSCs have emerged as the possible main player in cancer for the resistance to conventional therapies. Indeed, CSCs exhibit a quiescent state characterized by slow cell cycling. Thus, it is possible that, unlike the highly proliferating cells, the CSC subpopulations may not be eradicated by chemotherapy and radiotherapy, causing the relapse of the disease. This hypothesis is reinforced by the observation by Giuffrida et al. [100] who treated thyroid CSC-enriched spheres with different chemotherapy drugs, including bortezomib, taxol, cisplatin, etoposide, doxorubicin and vincristine. In those experiments, CSCs were more resistant than their parental differentiated PTC cells to chemotherapy [100]. Li and colleagues [101] have reported an increase in resistance to cisplatin in SP cells of various thyroid cancer lines compared with non-SP cells. Similarly, the CD133+ CSC population isolated from both human ATC cell lines and primary patient ATC specimens (ATC-CD133+ cells) displayed enhanced resistance to conventional chemotherapeutic drugs and ionizing radiation as compared to a differentiated non-CSC population (ATC-CD133−). Interestingly, the inhibition of a pivotal factor for the self-renewal of thyroid CSCs, STAT3 signaling, with cucurbitacin I significantly increased sensitivity to both radiotherapy and chemotherapy and suppressed self-renewing abilities by inducing apoptosis in ATC-CD133+ cells [82]. Thus, the eradication of CSC populations may significantly reduce the therapy-resistant phenotype and prevent tumor relapse.

### 6.2. Over-Expression of ATP-Binding Cassette

It is well-known that CSCs have an increased ability to extrude drugs via several multidrug resistance-related ABC transporters, including ABCB1/P-gp and ABCG2/BCRP, which confer drug resistance to the cell. A significant increase in the SP has been detected in a doxorubicin-resistant thyroid cancer cell subline, Hth74R, enriched with Oct4-positive cancer stem-like cells with respect to the parental Hth74 cell line. Furthermore, pharmacological inhibition of ABC transporters restored the sensitivity of Hth74R cells to doxorubicin [20]. Carina and colleagues [102] also reported that the silencing of SOX2 sensitized ATC-CSCs to chemotherapeutic agents by directly suppressing ABCG2. Whether these data suggest that the over-expression of ABC transporter proteins is probably one of the most important mechanisms of CSC-mediated resistance to conventional drugs in thyroid cancer, their potential impact in the therapeutic outcome of KIs in thyroid cancers has not yet been adequately explored. Unfortunately, it is still unknown whether chronic administration of KIs leads to CSC phenotypes characterized by ABC transporter’s up-regulation and reduced cell accumulation of drugs that result in acquired resistance to therapies and tumor relapse. However, it is reasonable to speculate that the intrinsic over-expression of ABC transporters in thyroid CSCs may explain the poor response to KIs in patients with thyroid cancer, but the underlying mechanisms among transporters, thyroid CSCs and KIs need to be determined.

### 6.3. Dysregulation of Growth Signaling Pathways of CSCs

One of the most important mechanisms by which CSCs survive cancer treatment is represented by deregulation of signaling pathways involved in stem cell self-renewal [103]. The activation of growth and survival signaling pathways, such as Hedgehog, Notch, JAK/STAT and Wnt/β-catenin, together with transcriptional regulators, including OCT4, SOX2 and YAP/TAZ, play a significant role in the expansion of CSCs and hence the resistance to therapy. Unfortunately, few reports have been published concerning the therapy resistance of thyroid CSCs. In particular, Heiden et al. [104] reported that the Sonic Hedgehog (Shh) signaling pathway, which is required for self-renewal of CSCs, is deregulated in ATC cell lines by Gli1-mediated up-regulation of Snail. Moreover, inhibition of the Shh pathway enhanced the radiation sensitivity of ATC CSCs, as well as impaired their self-renewal capacity [104]. Carina and colleagues [102] observed that the knockdown of SOX2 enhanced doxorubicin and cisplatin sensitivity via the inhibition of ABCG2 expression in SW1736, a thyroid cancer cell line with enriched CSC phenotypes. Finally, the inhibition of STAT3 signaling by cucurbitacin I was found to cause a decrease in CSC-like properties, and thereby sensitized ATC-CD133+ cells to chemoradiotherapy [82]. Similarly, curcumin has been observed to promote apoptosis and enhance the anticancer activity of cisplatin in thyroid CSCs by targeting STAT3 signaling [105]. Although no experimental data are available for thyroid cancer, it is likely the activation of growth signaling pathways in thyroid CSCs may have a considerable impact on the response of kinase inhibitors as reported for others cancers [106,107]. Taken together, these studies endorse the existing relationship between CSCs and resistance to therapies, suggesting that targeting CSCs could be a promising approach to overcoming resistance.

### 6.4. Activation of the Bypass Pathways

Another factor contributing to the therapy resistance of CSCs is the activation of the bypass pathways to therapeutic insult. Our recent report indicates that treatment with the BRAF inhibitor vemurafenib causes a paradoxical stimulatory effect on mutant BRAF V600E in thyroid cancer stem cells, differing from their parental monolayer cultures. Indeed, while the of BRAFV600E activity inhibition by vemurafenib resulted in decreased ERK phosphorylation and inhibition of cell growth in a mutant BRAFV600E whole thyroid cancer cell population, it induced a re-activation of ERK and a progressive increase in AKT activation in a CSC subpopulation. Importantly, we found that the resistance of thyroid CSCs to vemurafenib depends on the higher MAP3K8 expression. Indeed, pharmacological inhibition of MAP3K8 sensitizes thyroid BRAFV600E mutant CSCs to treatment with vemurafenib, suggesting therefore that this cell subpopulation can be involved in the failure of the therapies targeting BRAFV600E in advanced thyroid cancer [108].

### 6.5. Phenotypic Alterations

EMT has also been shown to promote resistance to treatment with kinase inhibitors, establishing a link with the resistant phenotype of CSCs [109]. As discussed in the above paragraphs, during EMT, epithelial cells lose their junctions and apical-basal polarity and acquire an invasive phenotype, as well as stemness properties. In a recent report, Lee et al. [110] identified a potential mechanism of EMT induction mediated by the activation of FGFR signaling, which was more pronounced in ATC compared to PTC patient-derived thyroid cancer cell lines. Interestingly, combined treatment with lenvatinib (in contrast to sorafenib), a multi-target kinase inhibitor of FGFR 1–4, and HNHA, a histone deacetylase inhibitor, triggers additive/synergistic anti-tumor response in patient-derived thyroid cancer cell lines. The combined treatment induced higher rates of apoptosis compared to each single compound by the suppression of EMT induction via FGFR signaling. Similar results were obtained in vivo in an orthotopic xenograft mouse model [110]. In addition, Dima et al. [111] observed that the treatment with crizotinib, a dual ALK/c-MET inhibitor, with additional activity on ROS1 kinase (c-ros), was less effective in thyroid CSCs overexpressing EMT-related genes than in their monolayer counterpart. The limited cell response to crizotinib may probably be associated with increased levels of RTK-related EMT phosphorylation observed in CSCs upon treatment with the inhibitor [111]. Collectively, these studies point to a reciprocal relationship between CSCs and EMT that could determine the development of drug resistance in advanced thyroid cancer.

### 6.6. Non-Coding RNAs and Exosome-Based Mechanisms

At the same time, many non-coding RNAs (ncRNAs), including long non-coding RNAs (lncRNAs), are reported to promote drug resistance by regulating the EMT process and specific signaling pathways in CSCs [112]. LncRNAs are non-protein coding transcripts longer than 200 nucleotides, which play crucial roles in regulating gene expression at transcriptional, post-transcriptional and epigenetic levels [113]. The aberrantly expressed lncRNAs have been implicated in the development, progression and resistance to therapy of various cancers, including thyroid cancer, regulating even EMT and cancer cell stemness [114,115,116,117]. Although the roles of lncRNA in thyroid CSCs are not fully understood, studies have revealed that several lncRNAs are involved in the regulation of CSCs in thyroid cancer. For instance, the lncRNA FOXD2 adjacent opposite strand RNA 1 (FOXD2-AS1) was found to be up-regulated in thyroid cancer tissue, and a higher expression was associated with poor recurrence-free survival in patients with DTC. In addition, the knockdown of FOXD2-AS1 suppressed the CSC-like phenotype and anoikis resistance of thyroid cancer cells through sponging mir-7-5p with consequent increase of telomerase reverse transcriptase (TERT) expression [118]. Interestingly, mir-7-5p has been characterized as a tumor suppressor in various cancers, regulating the differentiation and EMT processes, and its over-expression significantly reduced the proliferation, migration and invasion of sorafenib-resistant cells in human hepatocellular carcinoma [119]. These data suggest that expression of FOXD2-AS1 and miR-7-5p may predict the outcomes/responses of patients to sorafenib.

Most importantly, the interactions of lncRNA with signaling pathways can regulate the therapy resistance property of thyroid CSCs. For example, papillary thyroid carcinoma susceptibility candidate 3 (PTCSC3) is a recently recognized tumor-suppressive lncRNA whose expression has been found to be strongly down-regulated in thyroid cancer tissue compared to healthy counterparts. PTCSC3 has been reported to regulate thyroid cancer cell proliferation and migration via modulating Wnt/β-catenin signaling [120]. Moreover, the forced re-introduction of PTCSC3 resulted in the suppression of stem cell properties and restored sensitivity to doxorubicin in ATC cells by regulating the STAT3/INO80 pathway, a well-known promoter of thyroid CSC growth [121,122].

BRAF-activated non-coding RNA (BANCR) has been observed to be over-expressed in cancer tissue compared with the adjacent normal tissue in patients with thyroid cancer, which promotes the growth of thyroid cancer cells and CSC-like phenotypic characteristics via the RAF/MEK/ERK signaling pathway [123]. Given that BANCR is strongly associated with BRAF V600E and the only approved treatment for patients carrying BRAF-mutated ATC is the combination of dabrafenib (BRAFi) and trametinib (MEKi), the role of BANCR as a possible valid biomarker for monitoring and predicting responsiveness to inhibitors could be investigated.

Accumulating evidence has suggested that the well-described oncogene nuclear enriched abundant transcript 1 (NEAT1) plays a significant role in the therapy resistance of various tumors, including thyroid cancer [124]. Yan and colleagues [124] have reported an increased expression of NEAT1 in ATC tissue and cell lines, and the silencing of NEAT1 sensitized ATC cells to cisplatin and enhances apoptosis by suppressing miR-9-5p sponging and regulating sperm-associated antigen 9 (SPAG9). Recently, higher NEAT1 expression was noted in sorafenib-resistant hepatocellular cancer cells, and its anti-sorafenib activity has been observed to be promoted by modulating miR-335 expression, which in turn regulates the activity of c-MET/AKT signaling [125]. As mentioned above, c-MET/AKT signaling is one of the most important mechanisms in the self-renewal of CSCs in thyroid cancer, suggesting that the over-expression of NEAT1 in ATC could explain the limited efficacy of sorafenib for the treatment of patients with ATC. So far, the eventual role and mechanism of how NEAT1 regulates resistance to KIs in thyroid cancer CSCs remain unknown.

Interestingly, lncRNAs can also be packaged into exosomes, a subset of multi-vesicular bodies (MVB), and act as modulators in cell–cell communication [126]. In a recent study, Hardin et al. [127] observed that exosomes from thyroid CSCs induced EMT in non-cancerous thyroid cells by the transfer and expression of the lncRNAs MALAT1 and linc-ROR, as well as the EMT marker SLUG and the stem cell transcription factor SOX2. Moreover, the exosomes of the CSCs significantly increased the proliferation and invasiveness of the treated normal thyroid cells compared to the control cells [127]. These data would suggest that the intercellular cross-talk mediated by the exosomes of the CSCs and their content could significantly contribute to the production of different subpopulations of cells in a tumor microenvironment characterized by a different phenotype (i.e., resistant to treatment) [128,129].

## 7. CSC-Based Therapy

The ability of CSCs to be resistant to current anti-cancer therapies, suggests the needed development of novel and effective therapeutic strategies to eradicate CSCs specifically. A promising strategy could be the inhibition of specific CSC-generating and CSC-expanding pathways, such as the Wnt, Shh, JAK/STAT3 and PI3/AKT signaling pathways, which regulate thyroid CSC self-renewal and tumor growth. Although encouraging preclinical findings have suggested that the agents that inhibit CSC pathways suppress thyroid cancer cell growth and indeed enhance cellular sensitivity to both targeted and conventional therapy. Unfortunately, there are no ongoing clinical trials targeting CSCs in advanced thyroid cancer. The inhibition of mTOR, a component of the PI3K/AKT signaling pathway, has shown limited activity in advanced or metastatic thyroid cancer [130], while the effects of other inhibitors of CSC-regulating pathways have not yet been evaluated [131]. It is important to note that the alternative signaling through pathways cross-talk and the multiple heterogeneous phenotypes of CSCs could complicate the development of effective CSC-targeting therapy for an individual patient. Recently, a high-throughput siRNA screen was conducted by Shiraiwa and colleagues [132] using a library collection of 719 human kinases to identify specific regulators of thyroid CSCs. The authors found that both JAK/STAT3 and NF-KB signaling pathways regulate the properties of CSCs in ATC cells, and combined treatment with their specific pharmacological inhibitors triggers an additive/synergistic anti-tumor response in ATC sphere formation, speculating the cross-talk between the STAT3 and NF-KB signaling pathways [132]. Thus, targeting these signaling pathways may be a promising therapeutic approach to treating patients with advanced thyroid cancer.

Finally, the effective eradication of CSCs will require a better understanding of the properties of CSCs, including lncRNA and exosome-mediated cell-cell communication. Several lncRNAs have been shown to contribute to the mechanisms of anti-cancer therapy resistance in thyroid CSCs. However, further research is needed to determine whether lncRNAs may represent a therapeutic approach to overcome resistance in thyroid cancer. Interestingly, modified exosomes delivering therapeutic agents, such as pro-apoptotic proteins, ncRNAs, and drugs could be used to treat cancer. Thus, the understanding of resistance mechanisms involving exosome-mediated cell-to-cell communication needs to be addressed by future therapeutic strategies for advanced thyroid cancer.

## 8. Conclusions

The vast majority of thyroid carcinomas are cured well by surgery and radioiodine, while a minor but consistent group are refractory to radioiodine therapy and, unfortunately, very resistant to conventional chemotherapy. This carcinoma subgroup is often progressive and may cause patient death in the short term. Molecular studies identifying mutations driving thyroid cancer progression allowed the use of selective inhibitors targeted to deregulated pathways. To date, however, these drugs have not provided any significant and durable effect, and their actions are often cytostatic rather than toxic. In addition, selective inhibitor use in the long term often results in the progressive development of tumor resistance and sometimes in impressive tumor dedifferentiation and relapse.

Recently, the identification of normal stem cells in human thyroid and, more specifically, cancer stem cells within thyroid carcinomas has shed light onto a possible mechanism of thyroid cancer progression, dedifferentiation, relapse and chemoresistance due to their great ability for DNA repair and sustaining epithelial to mesenchymal transition. Hence, understanding the mechanisms regulating thyroid cancer stem cell proliferation and survival may provide valuable tools for tumor targeting that may be adjuvant in the treatment of aggressive thyroid carcinomas. In this light, studies aimed at describing pathways specifically activated in thyroid cancer stem cells and responsible for the interaction with the stem cell niche microenvironment are useful to developing novel drugs for these lethal tumors. Moreover, evaluation of the mechanisms underlying the differentiation process of both normal and cancer thyroid stem cells may prove useful for tailoring re-differentiating therapies aimed at restoring radioiodine uptake in less differentiated forms. It is also reasonable to suppose that isolation of thyroid cancer stem cells ex vivo after tumor surgery and debulking may provide a useful preclinical model for testing more selective and effective drugs.

Although these are promising and intriguing results, several issues and limitations should be better addressed. A comparative analysis of the characteristics of CSCs from different types of tumors and thyroid carcinomas has not yet been performed. Moreover, the in vivo models used in these studies do not recapitulate the biological complexity of tumors. Most studies on CSCs in general, and in thyroid CSCs in particular, addressing the ability to form tumors and to respond to therapies were performed in immune-deficient mice, in the absence of an active immune response. It should also be taken into account that thyroid CSCs live in a specific niche that sustains their survival. Most studies were performed with isolated CSCs devoid of the microenvironment present in the whole patient. Hence, the environmental factors in thyroid CSC niches that could influence the response to the different specific therapies are not well understood and should be studied in detail. A limit to studying isolated thyroid CSCs is also to ignore the possibility that CSCs share some signaling pathways with normal stem cells in vivo. Hence, not all the regulatory pathways that contribute to the biology of CSCs are appropriate targets in cancer treatment because of the important side effects. Another open question is whether CSCs should be activated or arrested by cancer therapy, because this action is dependent on tumor type and histotype and may be crucial for the restoration of RAI uptake. In conclusion, despite the compelling evidence in this field, targeting thyroid cancer stem cells at bedside is not available to date. However, this therapeutic option remains a useful possibility to prevent tumor relapse and progression, as well as to restore RAI uptake. Clinical trials in this direction should be encouraged.

## References

[1] Howlader N., Noone A.M., Krapcho M., Miller D., Bishop K., Altekruse S.F., Kosary C.L., Yu M., Ruhl J., Tatalovich Z., Bethesda M.D. (2015). SEER Cancer Statistics Review, 1975–2013.

[2] Vaccarella S., Franceschi S., Bray F., Wild C.P., Plummer M., Dal Maso L. (2016). Worldwide Thyroid-Cancer Epidemic? The Increasing Impact of Overdiagnosis. N. Engl. J. Med..

[3] Lim H., Devesa S.S., Sosa J.A., Check D., Kitahara C.M. (2017). Trends in Thyroid Cancer Incidence and Mortality in the United States, 1974–2013. JAMA.

[4] Pellegriti G., Frasca F., Regalbuto C., Squatrito S., Vigneri R. (2013). Worldwide Increasing Incidence of Thyroid Cancer: Update on Epidemiology and Risk Factors. J. Cancer Epidemiol..

[5] Nixon I.J., Suarez C., Simo R., Sanabria A., Angelos P., Rinaldo A., Rodrigo J.P., Kowalski L.P., Hartl D.M., Hinni M.L. (2016). The Impact of Family History on Non-Medullary Thyroid Cancer. Eur. J. Surg. Oncol..

[6] Jemal A., Ward E.M., Johnson C.J., Cronin K.A., Ma J., Ryerson B., Mariotto A., Lake A.J., Wilson R., Sherman R.L. (2017). Annual Report to the Nation on the Status of Cancer, 1975–2014, Featuring Survival. J. Natl. Cancer Inst..

[7] Durante C., Haddy N., Baudin E., Leboulleux S., Hartl D., Travagli J.P., Caillou B., Ricard M., Lumbroso J.D., De Vathaire F. (2006). Long-Term Outcome of 444 Patients with Distant Metastases from Papillary and Follicular Thyroid Carcinoma: Benefits and Limits of Radioiodine Therapy. J. Clin. Endocrinol. Metab..

[8] Saini S., Tulla K., Maker A.V., Burman K.D., Prabhakar B.S. (2018). Therapeutic Advances in Anaplastic Thyroid Cancer: A Current Perspective. Mol. Cancer.

[9] Albero A., Lopez J.E., Torres A., de la Cruz L., Martin T. (2016). Effectiveness of Chemotherapy in Advanced Differentiated Thyroid Cancer: A Systematic Review. Endocr. Relat. Cancer.

[10] Frampton J.E. (2016). Lenvatinib: A Review in Refractory Thyroid Cancer. Target. Oncol..

[11] Manfredi G.I., Dicitore A., Gaudenzi G., Caraglia M., Persani L., Vitale G. (2015). PI3K/Akt/mTOR Signaling in Medullary Thyroid Cancer: A Promising Molecular Target for Cancer Therapy. Endocrine.

[12] Abdel-Rahman O. (2015). Targeting Vascular Endothelial Growth Factor (VEGF) Pathway in Iodine-Refractory Differentiated Thyroid Carcinoma (DTC): From Bench to Bedside. Crit. Rev. Oncol. Hematol..

[13] Brose M.S., Nutting C.M., Jarzab B., Elisei R., Siena S., Bastholt L., de la Fouchardiere C., Pacini F., Paschke R., Shong Y.K. (2014). Sorafenib in Radioactive Iodine-Refractory, Locally Advanced or Metastatic Differentiated Thyroid Cancer: A Randomised, Double-Blind, Phase 3 Trial. Lancet.

[14] Schlumberger M., Tahara M., Wirth L.J., Robinson B., Brose M.S., Elisei R., Habra M.A., Newbold K., Shah M.H., Hoff A.O. (2015). Lenvatinib Versus Placebo in Radioiodine-Refractory Thyroid Cancer. N. Engl. J. Med..

[15] Asakawa H., Kobayashi T., Komoike Y., Maruyama H., Nakano Y., Tamaki Y., Matsuzawa Y., Monden M. (1997). Chemosensitivity of Anaplastic Thyroid Carcinoma and Poorly Differentiated Thyroid Carcinoma. Anticancer Res..

[16] Sugawara I., Masunaga A., Itoyama S., Sumizawa T., Akiyama S., Yamashita T. (1995). Expression of Multidrug Resistance-Associated Protein (MRP) in Thyroid Cancers. Cancer Lett..

[17] Wang S.H., Phelps E., Utsugi S., Baker J.R. (2001). Susceptibility of Thyroid Cancer Cells to 7-Hydroxystaurosporine-Induced Apoptosis Correlates with Bcl-2 Protein Level. Thyroid.

[18] Saylor P.J., Escudier B., Michaelson M.D. (2012). Importance of Fibroblast Growth Factor Receptor in Neovascularization and Tumor Escape from Antiangiogenic Therapy. Clin. Genitourin. Cancer.

[19] Monteiro J., Fodde R. (2010). Cancer Stemness and Metastasis: Therapeutic Consequences and Perspectives. Eur. J. Cancer.

[20] Zheng X., Cui D., Xu S., Brabant G., Derwahl M. (2010). Doxorubicin Fails to Eradicate Cancer Stem Cells Derived from Anaplastic Thyroid Carcinoma Cells: Characterization of Resistant Cells. Int. J. Oncol..

[21] Smallridge R.C. (2012). Approach to the Patient with Anaplastic Thyroid Carcinoma. J. Clin. Endocrinol. Metab..

[22] Marotta V., Sciammarella C., Vitale M., Colao A., Faggiano A. (2015). The evolving field of kinase inhibitors in thyroid cancer. Crit. Rev. Oncol. Hematol..

[23] Cancer Genome Atlas Research Network (2014). Integrated Genomic Characterization of Papillary Thyroid Carcinoma. Cell.

[24] Giordano T.J. (2016). Follicular Cell Thyroid Neoplasia: Insights from Genomics and The Cancer Genome Atlas Research Network. Curr. Opin. Oncol..

[25] Santoro M., Melillo R.M. (2015). Genetics: The Genomic Landscape of Papillary Thyroid Carcinoma. Nat. Rev. Endocrinol..

[26] Raman P., Koenig R.J. (2014). Pax-8-PPAR-Gamma Fusion Protein in Thyroid Carcinoma. Nat. Rev. Endocrinol..

[27] Melo M., da Rocha A.G., Vinagre J., Batista R., Peixoto J., Tavares C., Celestino R., Almeida A., Salgado C., Eloy C. (2014). TERT Promoter Mutations are a Major Indicator of Poor Outcome in Differentiated Thyroid Carcinomas. J. Clin. Endocrinol. Metab..

[28] Landa I., Ibrahimpasic T., Boucai L., Sinha R., Knauf J.A., Shah R.H., Dogan S., Ricarte-Filho J.C., Krishnamoorthy G.P., Xu B. (2016). Genomic and Transcriptomic Hallmarks of Poorly Differentiated and Anaplastic Thyroid Cancers. J. Clin. Investig..

[29] Ciampi R., Mian C., Fugazzola L., Cosci B., Romei C., Barollo S., Cirello V., Bottici V., Marconcini G., Rosa P.M. (2013). Evidence of a Low Prevalence of RAS Mutations in a Large Medullary Thyroid Cancer Series. Thyroid.

[30] Wells S.A., Asa S.L., Dralle H., Elisei R., Evans D.B., Gagel R.F., Lee N., Machens A., Moley J.F., Pacini F. (2015). Revised American Thyroid Association Guidelines for the Management of Medullary Thyroid Carcinoma. Thyroid.

[31] Kondo T., Ezzat S., Asa S.L. (2006). Pathogenetic Mechanisms in Thyroid Follicular-Cell Neoplasia. Nat. Rev. Cancer.

[32] Xing M. (2013). Molecular Pathogenesis and Mechanisms of Thyroid Cancer. Nat. Rev. Cancer.

[33] Kim D.S., McCabe C.J., Buchanan M.A., Watkinson J.C. (2003). Oncogenes in Thyroid Cancer. Clin. Otolaryngol. Allied Sci..

[34] Fagin J.A., Mitsiades N. (2008). Molecular Pathology of Thyroid Cancer: Diagnostic and Clinical Implications. Best Pract. Res. Clin. Endocrinol. Metab..

[35] Nikiforova M.N., Nikiforov Y.E. (2008). Molecular Genetics of Thyroid Cancer: Implications for Diagnosis, Treatment and Prognosis. Expert Rev. Mol. Diagn..

[36] Suarez H.G. (2000). Molecular Basis of Epithelial Thyroid Tumorigenesis. C. R. Acad. Sci..

[37] Bonnet D., Dick J.E. (1997). Human Acute Myeloid Leukemia is Organized as a Hierarchy that Originates from a Primitive Hematopoietic Cell. Nat. Med..

[38] Pardal R., Clarke M.F., Morrison S.J. (2003). Applying the Principles of Stem-Cell Biology to Cancer. Nat. Rev. Cancer.

[39] Polyak K., Hahn W.C. (2006). Roots and Stems: Stem Cells in Cancer. Nat. Med..

[40] Zhang P., Zuo H., Ozaki T., Nakagomi N., Kakudo K. (2006). Cancer Stem Cell Hypothesis in Thyroid Cancer. Pathol. Int..

[41] Takano T., Amino N. (2005). Fetal Cell Carcinogenesis: A New Hypothesis for Better Understanding of Thyroid Carcinoma. Thyroid.

[42] Zito G., Richiusa P., Bommarito A., Carissimi E., Russo L., Coppola A., Zerilli M., Rodolico V., Criscimanna A., Amato M. (2008). In Vitro Identification and Characterization of CD133(pos) Cancer Stem-Like Cells in Anaplastic Thyroid Carcinoma Cell Lines. PLoS ONE.

[43] Klonisch T., Hoang-Vu C., Hombach-Klonisch S. (2009). Thyroid Stem Cells and Cancer. Thyroid.

[44] Fierabracci A., Puglisi M.A., Giuliani L., Mattarocci S., Gallinella-Muzi M. (2008). Identification of an Adult Stem/Progenitor Cell-Like Population in the Human Thyroid. J. Endocrinol..

[45] Dumont J.E., Lamy F., Roger P., Maenhaut C. (1992). Physiological and Pathological Regulation of Thyroid Cell Proliferation and Differentiation by Thyrotropin and Other Factors. Physiol. Rev..

[46] Thomas T., Nowka K., Lan L., Derwahl M. (2006). Expression of Endoderm Stem Cell Markers: Evidence for the Presence of Adult Stem Cells in Human Thyroid Glands. Thyroid.

[47] Harach H.R., Vujanic G.M., Jasani B. (1993). Ultimobranchial Body Nests in Human Fetal Thyroid: An Autopsy, Histological, and Immunohistochemical Study in Relation to Solid Cell Nests and Mucoepidermoid Carcinoma of the Thyroid. J. Pathol..

[48] Cameselle-Teijeiro J., Febles-Perez C., Sobrinho-Simoes M. (1995). Papillary and mucoepidermoid carcinoma of the thyroid with anaplastic transformation: A case report with histologic and immunohistochemical findings that support a provocative histogenetic hypothesis. Pathol. Res. Pract..

[49] Reis-Filho J.S., Preto A., Soares P., Ricardo S., Cameselle-Teijeiro J., Sobrinho-Simoes M. (2003). p63 Expression in Solid Cell Nests of the Thyroid: Further Evidence for a Stem Cell Origin. Mod. Pathol..

[50] Campagnoli C., Roberts I., Kumar S., Bennett P.R., Fisk N.M. (2001). Clonal Culture of Fetal Cells from Maternal Blood. Lancet.

[51] Sanders J.E., Hoffmeister P.A., Woolfrey A.E., Carpenter P.A., Storer B.E., Storb R.F., Appelbaum F.R. (2009). Thyroid Function Following Hematopoietic Cell Transplantation in Children: 30 Years’ Experience. Blood.

[52] Hoshi N., Kusakabe T., Taylor B.J., Kimura S. (2007). Side Population Cells in the Mouse Thyroid Exhibit Stem/Progenitor Cell-Like Characteristics. Endocrinology.

[53] Lan L., Cui D., Nowka K., Derwahl M. (2007). Stem Cells Derived from Goiters in Adults Form Spheres in Response to Intense Growth Stimulation and Require Thyrotropin for Differentiation into Thyrocytes. J. Clin. Endocrinol. Metab..

[54] Malaguarnera R., Frasca F., Garozzo A., Giani F., Pandini G., Vella V., Vigneri R., Belfiore A. (2011). Insulin Receptor Isoforms and Insulin-Like Growth Factor Receptor in Human Follicular Cell Precursors from Papillary Thyroid Cancer and Normal Thyroid. J. Clin. Endocrinol. Metab..

[55] Kimura S. (2014). Thyroid Regeneration: How Stem Cells Play a Role?. Front. Endocrinol..

[56] Takano T., Amino N. (2002). Cancer-Specific mRNAs in Thyroid Carcinomas: Detection, Use, and Their Implication in Thyroid Carcinogenesis. Endocr. J..

[57] Takano T. (2014). Fetal Cell Carcinogenesis of the Thyroid: A Modified Theory Based on Recent Evidence. Endocr. J..

[58] Jhiang S.M., Sagartz J.E., Tong Q., Parker-Thornburg J., Capen C.C., Cho J.Y., Xing S., Ledent C. (1996). Targeted Expression of the ret/PTC1 Oncogene Induces Papillary Thyroid Carcinomas. Endocrinology.

[59] Charles R.P., Iezza G., Amendola E., Dankort D., McMahon M. (2011). Mutationally Activated BRAF(V600E) Elicits Papillary Thyroid Cancer in the Adult Mouse. Cancer Res..

[60] Shimamura M., Nakahara M., Orim F., Kurashige T., Mitsutake N., Nakashima M., Kondo S., Yamada M., Taguchi R., Kimura S. (2013). Postnatal Expression of BRAFV600E Does Not Induce Thyroid Cancer in Mouse Models of Thyroid Papillary Carcinoma. Endocrinology.

[61] Todaro M., Iovino F., Eterno V., Cammareri P., Gambara G., Espina V., Gulotta G., Dieli F., Giordano S., De Maria R. (2010). Tumorigenic and Metastatic Activity of Human Thyroid Cancer Stem Cells. Cancer Res..

[62] Gibelli B., El-Fattah A., Giugliano G., Proh M., Grosso E. (2009). Thyroid Stem Cells—Danger or Resource?. Acta Otorhinolaryngol. Ital..

[63] Reya T., Morrison S.J., Clarke M.F., Weissman I.L. (2001). Stem Cells, Cancer, and Cancer Stem Cells. Nature.

[64] Wicha M.S., Liu S., Dontu G. (2006). Cancer Stem Cells: An Old Idea—A Paradigm Shift. Cancer Res..

[65] Sell S. (2004). Stem Cell Origin of Cancer and Differentiation Therapy. Crit. Rev. Oncol. Hematol..

[66] Rosen J.M., Jordan C.T. (2009). The Increasing Complexity of the Cancer Stem Cell Paradigm. Science.

[67] Jordan C.T., Guzman M.L., Noble M. (2006). Cancer Stem Cells. N. Engl. J. Med..

[68] Deng S., Yang X., Lassus H., Liang S., Kaur S., Ye Q., Li C., Wang L.P., Roby K.F., Orsulic S. (2010). Distinct Expression Levels and Patterns of Stem Cell Marker, Aldehyde Dehydrogenase Isoform 1 (ALDH1), in Human Epithelial Cancers. PLoS ONE.

[69] Kimlin L., Virador V. (2013). Cellular Populations Isolated from Newborn Mouse Skin Including Mesenchymal Stem Cells. Methods Mol. Biol..

[70] Weiswald L.B., Bellet D., Dangles-Marie V. (2015). Spherical Cancer Models in Tumor Biology. Neoplasia.

[71] Hardin H., Montemayor-Garcia C., Lloyd R.V. (2013). Thyroid Cancer Stem-Like Cells and Epithelial-Mesenchymal Transition in Thyroid Cancers. Hum. Pathol..

[72] Lan L., Luo Y., Cui D., Shi B.Y., Deng W., Huo L.L., Chen H.L., Zhang G.Y., Deng L.L. (2013). Epithelial- Mesenchymal Transition Triggers Cancer Stem Cell Generation in Human Thyroid Cancer Cells. Int. J. Oncol..

[73] Varjosalo M., Taipale J. (2008). Hedgehog: Functions and Mechanisms. Genes Dev..

[74] Fan X., Matsui W., Khaki L., Stearns D., Chun J., Li Y.M., Eberhart C.G. (2006). Notch Pathway Inhibition Depletes Stem-Like Cells and Blocks Engraftment in Embryonal Brain Tumors. Cancer Res..

[75] Luo M., Zhao X., Chen S., Liu S., Wicha M.S., Guan J.L. (2013). Distinct FAK Activities Determine Progenitor and Mammary Stem Cell Characteristics. Cancer Res..

[76] Van Amerongen R., Bowman A.N., Nusse R. (2012). Developmental Stage and Time Dictate the Fate of Wnt/Beta-Catenin-Responsive Stem Cells in the Mammary Gland. Cell Stem Cell.

[77] Kamran M.Z., Patil P., Gude R.P. (2013). Role of STAT3 in Cancer Metastasis and Translational Advances. Biomed. Res. Int..

[78] Wang M.L., Chiou S.H., Wu C.W. (2013). Targeting Cancer Stem Cells: Emerging Role of Nanog Transcription Factor. Onco. Targets Ther..

[79] Giani F., Vella V., Nicolosi M.L., Fierabracci A., Lotta S., Malaguarnera R., Belfiore A., Vigneri R., Frasca F. (2015). Thyrospheres From Normal or Malignant Thyroid Tissue Have Different Biological, Functional, and Genetic Features. J. Clin. Endocrinol. Metab..

[80] Han S.A., Jang J.H., Won K.Y., Lim S.J., Song J.Y. (2017). Prognostic Value of Putative Cancer Stem Cell Markers (CD24, CD44, CD133, and ALDH1) in Human Papillary Thyroid Carcinoma. Pathol. Res. Pract..

[81] Liotti F., Collina F., Pone E., La Sala L., Franco R., Prevete N., Melillo R.M. (2017). Interleukin-8, but not the Related Chemokine CXCL1, Sustains an Autocrine Circuit Necessary for the Properties and Functions of Thyroid Cancer Stem Cells. Stem Cells.

[82] Tseng L.M., Huang P.I., Chen Y.R., Chen Y.C., Chou Y.C., Chen Y.W., Chang Y.L., Hsu H.S., Lan Y.T., Chen K.H. (2012). Targeting Signal Transducer and Activator of Transcription 3 Pathway by Cucurbitacin I Diminishes Self-Renewing and Radiochemoresistant Abilities in Thyroid Cancer-Derived CD133+ Cells. J. Pharmacol. Exp. Ther..

[83] Belfiore A., Malaguarnera R., Vella V., Lawrence M.C., Sciacca L., Frasca F., Morrione A., Vigneri R. (2017). Insulin Receptor Isoforms in Physiology and Disease: An Updated View. Endocr. Rev..

[84] Vella V., Malaguarnera R. (2018). The Emerging Role of Insulin Receptor Isoforms in Thyroid Cancer: Clinical Implications and New Perspectives. Int. J. Mol. Sci..

[85] Vella V., Milluzzo A., Scalisi N.M., Vigneri P., Sciacca L. (2018). Insulin Receptor Isoforms in Cancer. Int. J. Mol. Sci..

[86] Ciavardelli D., Bellomo M., Consalvo A., Crescimanno C., Vella V. (2017). Metabolic Alterations of Thyroid Cancer as Potential Therapeutic Targets. Biomed. Res. Int..

[87] Vella V., Nicolosi M.L., Giuliano M., Morrione A., Malaguarnera R., Belfiore A. (2019). Insulin Receptor Isoform A Modulates Metabolic Reprogramming of Breast Cancer Cells in Response to IGF2 and Insulin Stimulation. Cells.

[88] Vella V., Malaguarnera R., Nicolosi M.L., Morrione A., Belfiore A. (2019). Insulin/IGF Signaling and Discoidin Domain Receptors: An Emerging Functional Connection. Biochim. Biophys. Acta Mol. Cell Res..

[89] Vella V., Malaguarnera R., Nicolosi M.L., Palladino C., Spoleti C., Massimino M., Vigneri P., Purrello M., Ragusa M., Morrione A. (2017). Discoidin Domain Receptor 1 Modulates Insulin Receptor Signaling and Biological Responses in Breast Cancer Cells. Oncotarget.

[90] Vella V., Nicolosi M.L., Cantafio P., Massimino M., Lappano R., Vigneri P., Ciuni R., Gangemi P., Morrione A., Malaguarnera R. (2019). DDR1 Regulates Thyroid Cancer Cell Differentiation via IGF-2/IR-A Autocrine Signaling Loop. Endocr. Relat. Cancer.

[91] Belfiore A., Malaguarnera R., Nicolosi M.L., Lappano R., Ragusa M., Morrione A., Vella V. (2018). A Novel Functional Crosstalk Between DDR1 and the IGF Axis and its Relevance for Breast Cancer. Cell Adh. Migr..

[92] Piscazzi A., Costantino E., Maddalena F., Natalicchio M.I., Gerardi A.M., Antonetti R., Cignarelli M., Landriscina M. (2012). Activation of the RAS/RAF/ERK Signaling Pathway Contributes to Resistance to Sunitinib in Thyroid Carcinoma Cell Lines. J. Clin. Endocrinol. Metab..

[93] Frasca F., Vella V., Nicolosi M.L., Messina R.L., Giani F., Lotta S., Vigneri P., Regalbuto C., Vigneri R. (2013). Thyroid Cancer Cell Resistance to Gefitinib Depends on the Constitutive Oncogenic Activation of the ERK Pathway. J. Clin. Endocrinol. Metab..

[94] Montero-Conde C., Ruiz-Llorente S., Dominguez J.M., Knauf J.A., Viale A., Sherman E.J., Ryder M., Ghossein R.A., Rosen N., Fagin J.A. (2013). Relief of Feedback Inhibition of HER3 Transcription by RAF and MEK Inhibitors Attenuates Their Antitumor Effects in BRAF-Mutant Thyroid Carcinomas. Cancer Discov..

[95] Byeon H.K., Na H.J., Yang Y.J., Kwon H.J., Chang J.W., Ban M.J., Kim W.S., Shin D.Y., Lee E.J., Koh Y.W. (2016). c-Met-Mediated Reactivation of PI3K/AKT Signaling Contributes to Insensitivity of BRAF(V600E) Mutant Thyroid Cancer to BRAF Inhibition. Mol. Carcinog..

[96] Danysh B.P., Rieger E.Y., Sinha D.K., Evers C.V., Cote G.J., Cabanillas M.E., Hofmann M.C. (2016). Long-Term Vemurafenib Treatment Drives Inhibitor Resistance Through a Spontaneous KRAS G12D Mutation in a BRAF V600E Papillary Thyroid Carcinoma Model. Oncotarget.

[97] Isham C.R., Netzel B.C., Bossou A.R., Milosevic D., Cradic K.W., Grebe S.K., Bible K.C. (2014). Development and Characterization of a Differentiated Thyroid Cancer Cell Line Resistant to VEGFR-Targeted Kinase Inhibitors. J. Clin. Endocrinol. Metab..

[98] Wagle N., Grabiner B.C., Van Allen E.M., Amin-Mansour A., Taylor-Weiner A., Rosenberg M., Gray N., Barletta J.A., Guo Y., Swanson S.J. (2014). Response and Acquired Resistance to Everolimus in Anaplastic Thyroid Cancer. N. Engl. J. Med..

[99] Prete A., Lo A.S., Sadow P.M., Bhasin S.S., Antonello Z.A., Vodopivec D.M., Ullas S., Sims J.N., Clohessy J., Dvorak A.M. (2018). Pericytes Elicit Resistance to Vemurafenib and Sorafenib Therapy in Thyroid Carcinoma via the TSP-1/TGFbeta1 Axis. Clin. Cancer Res..

[100] Giuffrida R., Adamo L., Iannolo G., Vicari L., Giuffrida D., Eramo A., Gulisano M., Memeo L., Conticello C. (2016). Resistance of Papillary Thyroid Cancer Stem Cells to Chemotherapy. Oncol. Lett..

[101] Li Z., Jiang X., Chen P., Wu X., Duan A., Qin Y. (2018). Combined Effects of Octreotide and Cisplatin on the Proliferation of Side Population Cells from Anaplastic Thyroid Cancer Cell Lines. Oncol. Lett..

[102] Carina V., Zito G., Pizzolanti G., Richiusa P., Criscimanna A., Rodolico V., Tomasello L., Pitrone M., Arancio W., Giordano C. (2013). Multiple Pluripotent Stem Cell Markers in Human Anaplastic Thyroid Cancer: The Putative Upstream Role of SOX2. Thyroid.

[103] Borah A., Raveendran S., Rochani A., Maekawa T., Kumar D.S. (2015). Targeting Self-Renewal Pathways in Cancer Stem Cells: Clinical Implications for Cancer Therapy. Oncogenesis.

[104] Heiden K.B., Williamson A.J., Doscas M.E., Ye J., Wang Y., Liu D., Xing M., Prinz R.A., Xu X. (2014). The Sonic Hedgehog Signaling Pathway Maintains the Cancer Stem Cell Self-Renewal of Anaplastic Thyroid Cancer by Inducing Snail Expression. J. Clin. Endocrinol. Metab..

[105] Khan A.Q., Ahmed E.I., Elareer N., Fathima H., Prabhu K.S., Siveen K.S., Kulinski M., Azizi F., Dermime S., Ahmad A. (2020). Curcumin-Mediated Apoptotic Cell Death in Papillary Thyroid Cancer and Cancer Stem-Like Cells through Targeting of the JAK/STAT3 Signaling Pathway. Int. J. Mol. Sci..

[106] Wang S., Cai L., Zhang F., Shang X., Xiao R., Zhou H. (2020). Inhibition of EZH2 Attenuates Sorafenib Resistance by Targeting NOTCH1 Activation-Dependent Liver Cancer Stem Cells via NOTCH1-Related MicroRNAs in Hepatocellular Carcinoma. Transl. Oncol..

[107] Bai X.Y., Zhang X.C., Yang S.Q., An S.J., Chen Z.H., Su J., Xie Z., Gou L.Y., Wu Y.L. (2016). Blockade of Hedgehog Signaling Synergistically Increases Sensitivity to Epidermal Growth Factor Receptor Tyrosine Kinase Inhibitors in Non-small-Cell Lung Cancer Cell Lines. PLoS ONE.

[108] Giani F., Russo G., Pennisi M., Sciacca L., Frasca F., Pappalardo F. (2019). Computational Modeling Reveals MAP3K8 as Mediator of Resistance to Vemurafenib in Thyroid Cancer Stem Cells. Bioinformatics.

[109] Singh A., Settleman J. (2010). EMT, Cancer Stem Cells and Drug Resistance: An Emerging Axis of Evil in the War on Cancer. Oncogene.

[110] Lee Y.S., Kim S.M., Kim B.W., Chang H.J., Kim S.Y., Park C.S., Park K.C., Chang H.S. (2018). Anti-Cancer Effects of HNHA and Lenvatinib by the Suppression of EMT-Mediated Drug Resistance in Cancer Stem Cells. Neoplasia.

[111] Dima M., Pecce V., Biffoni M., Di Gioia C.R., Tallini G., Biffoni M., Rosignolo F., Verrienti A., Sponziello M., Damante G. (2016). Molecular profiles of cancer stem-like cell populations in aggressive thyroid cancers. Endocrine.

[112] Heery R., Finn S.P., Cuffe S., Gray S.G. (2017). Long Non-Coding RNAs: Key Regulators of Epithelial-Mesenchymal Transition, Tumour Drug Resistance and Cancer Stem Cells. Cancers.

[113] Wang K.C., Chang H.Y. (2011). Molecular Mechanisms of Long Noncoding RNAs. Mol. Cell.

[114] Huo X., Han S., Wu G., Latchoumanin O., Zhou G., Hebbard L., George J., Qiao L. (2017). Dysregulated Long Noncoding RNAs (lncRNAs) in Hepatocellular Carcinoma: Implications for Tumorigenesis, Disease Progression, and Liver Cancer Stem Cells. Mol. Cancer.

[115] Peng X., Zhang K., Ma L., Xu J., Chang W. (2020). The Role of Long Non-Coding RNAs in Thyroid Cancer. Front. Oncol..

[116] Jiang W., Xia J., Xie S., Zou R., Pan S., Wang Z.W., Assaraf Y.G., Zhu X. (2020). Long Non-Coding RNAs as a Determinant of Cancer Drug Resistance: Towards the Overcoming of Chemoresistance via Modulation of lncRNAs. Drug Resist. Updates.

[117] Castro-Oropeza R., Melendez-Zajgla J., Maldonado V., Vazquez-Santillan K. (2018). The Emerging Role of lncRNAs in the Regulation of Cancer Stem Cells. Cell. Oncol..

[118] Liu X., Fu Q., Li S., Liang N., Li F., Li C., Sui C., Dionigi G., Sun H. (2019). LncRNA FOXD2-AS1 Functions as a Competing Endogenous RNA to Regulate TERT Expression by Sponging miR-7-5p in Thyroid Cancer. Front. Endocrinol..

[119] Kabir T.D., Ganda C., Brown R.M., Beveridge D.J., Richardson K.L., Chaturvedi V., Candy P., Epis M., Wintle L., Kalinowski F. (2018). A MicroRNA-7/Growth Arrest Specific 6/TYRO3 Axis Regulates the Growth and Invasiveness of Sorafenib-Resistant Cells in Human Hepatocellular Carcinoma. Hepatology.

[120] Wang X., Lu X., Geng Z., Yang G., Shi Y. (2017). LncRNA PTCSC3/miR-574-5p Governs Cell Proliferation and Migration of Papillary Thyroid Carcinoma via Wnt/β-Catenin Signaling. J. Cell. Biochem..

[121] Wang X., Liu Y., Fan Y., Liu Z., Yuan Q., Jia M., Geng Z., Gu L., Lu X. (2018). LncRNA PTCSC3 Affects Drug Resistance of Anaplastic Thyroid Cancer Through STAT3/INO80 Pathway. Cancer Biol. Ther..

[122] Sheng W., Chen Y., Gong Y., Dong T., Zhang B., Gao W. (2016). Mir-148a Inhibits Self-Renewal of Thyroid Cancer Stem Cells via Repressing ino80 Expression. Oncol. Rep..

[123] Wang Y.Y., Lin X.D., Fu X.H., Yan W., Lin F.S., Kuang P.H., Luo Y., Lin E., Hong X., Wu G. (2018). Long Non-Coding RNA BANCR Regulates Cancer Stem Cell Markers in Papillary Thyroid Cancer via the RAF/MEK/ERK Signaling Pathway. Oncol. Rep..

[124] Yan P., Su Z., Zhang Z., Gao T. (2019). LncRNA NEAT1 Enhances the Resistance of Anaplastic Thyroid Carcinoma Cells to Cisplatin by Sponging miR-9-5p and Regulating SPAG9 Expression. Int. J. Oncol..

[125] Niu Y., Tang G., Wu X., Wu C. (2020). LncRNA NEAT1 Modulates Sorafenib Resistance in Hepatocellular Carcinoma Through Regulating the miR-149-5p/AKT1 Axis. Saudi J. Gastroenterol..

[126] Valadi H., Ekstrom K., Bossios A., Sjostrand M., Lee J.J., Lotvall J.O. (2007). Exosome-Mediated Transfer of mRNAs and microRNAs is a Novel Mechanism of Genetic Exchange Between Cells. Nat. Cell Biol..

[127] Hardin H., Helein H., Meyer K., Robertson S., Zhang R., Zhong W., Lloyd R.V. (2018). Thyroid Cancer Stem-Like Cell Exosomes: Regulation of EMT via Transfer of lncRNAs. Lab. Investig..

[128] Sun Z., Wang L., Dong L., Wang X. (2018). Emerging Role of Exosome Signalling in Maintaining Cancer Stem Cell Dynamic Equilibrium. J. Cell Mol. Med..

[129] Xavier C.P.R., Caires H.R., Barbosa M.A.G., Bergantim R., Guimaraes J.E., Vasconcelos M.H. (2020). The Role of Extracellular Vesicles in the Hallmarks of Cancer and Drug Resistance. Cells.

[130] Lim S.M., Chang H., Yoon M.J., Hong Y.K., Kim H., Chung W.Y., Park C.S., Nam K.H., Kang S.W., Kim M.K. (2013). A Multicenter, Phase II Trial of Everolimus in Locally Advanced or Metastatic Thyroid Cancer of All Histologic Subtypes. Ann. Oncol..

[131] Tirrò E., Martorana F., Romano C., Vitale S.R., Motta G., Di Gregorio S., Massimino M., Pennisi M.S., Stella S., Puma A. (2019). Molecular Alterations in Thyroid Cancer: From Bench to Clinical Practice. Genes.

[132] Shiraiwa K., Matsuse M., Nakazawa Y., Ogi T., Suzuki K., Saenko V.A., Xu S., Umezawa K., Yamashita S., Tsukamoto K. (2019). JAK/STAT3 and NF-κB Signaling Pathways Regulate Cancer Stem-Cell Properties in Anaplastic Thyroid Cancer Cells. Thyroid.

